# The Fundamental Frequency of Voice as a Potential Stress Biomarker: A Systematic Review and Meta–Analysis

**DOI:** 10.1002/smi.70112

**Published:** 2025-10-16

**Authors:** Diogo de Lacerda Veiga, Thales Marcon Almeida, Ricardo Riyoiti Uchida, Quirino Cordeiro

**Affiliations:** ^1^ Mental Health Department Santa Casa de Sao Paulo School of Medical Sciences São Paulo Brazil; ^2^ Mental Health Department GRETAH—Anxiety and Mood Disorders Program Santa Casa de Sao Paulo School of Medical Sciences São Paulo Brazil

**Keywords:** acoustic parameters, fundamental frequency, noninvasive biomarkers, psychological stress, voice stress analysis

## Abstract

Stress alters vocal production, particularly by affecting laryngeal muscle function. Despite several studies on voice acoustics under stress, no systematic synthesis exists. This study aimed to conduct a systematic review and meta‐analysis of experimental studies assessing the impact of stress on vocal fundamental frequency (F0). Following PRISMA guidelines, a systematic search was conducted in PubMed and Scopus databases for studies published between January 2010 and September 2024. Eligible studies included adult participants exposed to experimental or naturalistic stressors, with pre‐ and post‐stress voice recordings analysed using objective acoustic measures. Data were extracted regarding F0, and random‐effects meta‐analyses were performed. Subgroup analyses were conducted based on gender, speech type, and stress induction method. Ten studies met the inclusion criteria, encompassing 1148 observations. The meta‐analysis showed a significant increase in F0 after stress (SMD = 0.5504, 95% CI [0.3014, 0.7995], *p* < 0.001) with substantial heterogeneity (*I* ^2^ = 69.2%). However, evidence of publication bias was observed; trim‐and‐fill attenuated the pooled estimate to a nonsignificant effect (SMD = 0.1710; 95% CI: −0.1472 to 0.4891; *p* = 0.2923). Subgroup analyses revealed stronger effects in women (SMD = 0.7128, 95% CI [0.3763, 1.0492], *p* < 0.001) and during spontaneous speech (SMD = 0.7911, 95% CI [0.4492, 1.1331], *p* < 0.001), with nonsignificant results for men and standardized speech. Effects tended to be larger for naturalistic stressors than validated laboratory procedures, though the between‐group comparison was not statistically significant. F0 shows promise for stress assessment, but publication bias and heterogeneity warrant caution. F0 should be considered a noninvasive candidate biomarker requiring validation in large, prospective studies using standardized protocols and representative, gender‐stratified cohorts.

## 1 Introduction

Stress can be understood as a disruption of homeodynamic balance triggered by real or perceived challenges, whether internal or external, commonly termed stressors. This state motivates a coordinated psychobiological response that engages the sympathetic nervous system (SNS) and the hypothalamic–pituitary–adrenal (HPA) axis (Agorastos and Chrousos 2022), which can alter respiratory and phonatory control (Van Puyvelde et al. 2018), motivating interest in voice as a noninvasive stress marker.

Voice, shaped by breathing, phonation, and resonance, conveys emotional and mental states (Van Puyvelde et al. 2018). Neurological control involves the limbic (emotional) and cortical motor (voluntary) pathways, from the primary motor cortex to the nucleus ambiguous via the vagus nerve (Splittgerber 2019). The vagus nerve is responsible for regulating speech tone and noise. The recurrent laryngeal nerves control the adductor and abductor muscles of the larynx, providing the necessary impulses to convert crude noise into speech. Additionally, the superior laryngeal and recurrent laryngeal nerves contract the cricothyroid and relax the vocal folds to adjust pitch (the perceptual correlate of fundamental frequency) (Câmara and Griessenauer 2015; Oxenham 2023). Vocal intensity varies according to vocal fold adduction and pulmonary pressure (Herbst et al. 2015; Zhang 2015). Stress is associated with physiological changes, particularly increased muscle tension in the phonatory system, especially in the cricothyroid muscle, and increased subglottic pressure during phonation, that may influence vocal tract dynamics and acoustic output (Van Puyvelde et al. 2018).

The acoustic parameters of speech are related to the stages of breathing, phonation, and resonance. In respiration, aspects such as syllables per phrase (SPP), inappropriate breathing pauses (IP), articulation rate (AR), and voice onset time (VOT) are analysed. In phonation, parameters include fundamental frequency (F0), which is the mean frequency of the vocal fold vibrations, its variability and range, timbre (harmonic richness factor, HRF), vocal cycle variations (VSSR), Jitter, Shimmer, and periodicity indices such as harmonic‐to‐noise ratio (HNR) and signal‐to‐noise ratio (SNR). In the resonance stage, the main measures are the formants (F1, F2, F3), as well as parameters derived from inverse filtering and cepstral analysis, such as Mel‐frequency cepstral coefficients (MFCCs), which reflect the quality of the vocal sound (Saggio and Costantini 2022; Van Puyvelde et al. 2018).

When stress activation becomes repeated or prolonged, the cumulative physiological burden (allostatic load) increases, contributing to multiple physical and psychiatric disorders, including depression, cardiovascular risk, and metabolic syndromes, underscoring the value of early detection and monitoring (Agorastos and Chrousos 2022; Guo et al. 2024; Kivimäki et al. 2023; Park et al. 2019). Stress can be assessed with self‐report scales (e.g., Perceived Stress Scale), behavioural observation (facial expressions, gestures, posture), and physiological indices such as salivary cortisol, the dexamethasone suppression test, heart rate variability, and skin conductance. However, these approaches have limitations: interviews and questionnaires are retrospective, time‐consuming, costly, and susceptible to cognitive bias and social desirability, while biomarker tests avoid self‐report bias; they often require invasive sampling or specialised equipment, limiting continuous, real‐world use (Alberdi et al. 2016; Bali and Jaggi 2015; Can et al. 2019; Giannakakis et al. 2022; Slavich et al. 2019). Noninvasive stress detection enables continuous, real‐world monitoring with high ecological validity, supporting early identification and timely interventions. Passive and contactless methods are more comfortable and safer, reducing the user's burden. Consequently, voice acoustic parameters are being investigated as noninvasive, passive, and scalable biomarkers of stress (Alberdi et al. 2016; Hassanpour and Yang 2025; Miller et al. 2024; Pinge et al. 2024; Rogan et al. 2024).

Despite the prevalence of stress in everyday contexts, multiple published studies use a standardized and validated laboratory protocols that reliably and validly induce stress, for example: the cold pressor test (CPT), with hand immersion in cold water (Fanninger et al. 2023), the Trier Social Stress Test (TSST), a kind of mock interview (Allen et al. 2017), and the Montreal Imaging Stress Task (MIST), an arithmetic task with feedback (Dedovic et al. 2005), among others (Bali and Jaggi 2015; Ferreira 2019).

While studies have reported associations between stress and changes in voice parameters such as F0, jitter, shimmer, HNR/SNR, these relationships remain largely correlational. Despite its potential as a noninvasive stress indicator (Kappen et al. 2023), voice analysis lacks a systematic meta‐analytic review, highlighting the need to integrate and critically assess the existing findings. The objective of this study is to conduct a systematic review and meta‐analysis of studies evaluating changes in vocal fundamental frequency (F0) induced by experimental stress models.

## 2 Methods

This study is a systematic review and meta‐analysis based on quantitative data. Study design and article selection followed the PICO framework (Population, Intervention, Comparison, Outcome), as listed below: (a) Population: Individuals subjected to stress; (b)Intervention: Exposure to stressors; (c) Comparison: Pre‐ and post‐stress vocal fundamental frequency; (d) Outcome: Changes in vocal fundamental frequency measured before and after stress.

### 2.1 Eligibility Criteria

This review included studies on adult participants (≥ 18 years) using objective acoustic measurements to quantify voice changes under stress. Eligible studies used validated scales or physiological measures to assess stress, included stress exposure, and provided a control or baseline comparison. And excluded studies without acoustic voice analysis under stress, lacking comparison groups, or involving participants with medical or psychiatric conditions affecting speech.

### 2.2 Study Identification

The PubMed and Scopus databases were searched for articles published between 2010 and September 2024. The PubMed search strategy was: ‘stress, psychological’ [MeSH Terms] OR ‘anxiety’ [MeSH Terms] OR ‘stress’ [All Fields] OR ‘stressed’ [All Fields] OR ‘stresses’ [All Fields] OR ‘stressful’ [All Fields] OR ‘stressfulness’ [All Fields] OR ‘stressing’ [All Fields] OR ‘stress detection’ [All Fields] AND ‘speech acoustics’ [MeSH Terms] OR ‘acoustic features’ [All Fields] OR ‘Harmonics‐to‐noise ratio’ [All Fields] OR ‘jitter’ [All Fields] OR ‘jittered’ [All Fields] OR ‘jittering’ [All Fields] OR ‘jitters’ [All Fields] OR ‘shimmer’ [All Fields] OR ‘shimmers’ [All Fields] OR ‘fundamental frequency’ [All Fields]. The Scopus search strategy was: ‘acoustic features’ OR ‘speech acoustics’ OR ‘Harmonics‐to‐noise ratio’ OR ‘jitter’ OR ‘Shimmer’ OR ‘fundamental frequency’ AND ‘psychological stress’ OR ‘stress’ OR ‘anxiety’ OR ‘stress detection’.

### 2.3 Study Selection

Two authors independently evaluated titles and abstracts based on pre‐established inclusion criteria. The Rayyan software (Ouzzani et al. 2016) was used for initial screening and duplicate detection. In case of duplicates, only the most complete manuscript was kept. Disagreements regarding inclusion were resolved by consensus among the authors.

### 2.4 Data Analysis

Data was extracted using a standardized form: author, year, study type, participant details, speech spontaneity, stress induction method, sample size, F0 means (pre/post stress), and standard deviations. The risk of bias was assessed using the ROBINS‐I (Risk Of Bias In Nonrandomised Studies ‐ of Interventions) tool for nonrandomised studies (Sterne et al. 2016). Statistical analysis was conducted using RStudio (version 1.2.5033.0) and R (version 3.6.2.27560), using meta‐analytic models to estimate effect sizes, assess heterogeneity, and explore potential bias. Extracted data were standardized to ensure compatibility of different measurement units. Random‐effects meta‐analyses were used to calculate combined effects. Effect sizes were expressed as standardized mean differences (SMD) with 95% confidence intervals (95% CI). Heterogeneity was assessed using: (a) Cochran's *Q* to detect significant heterogeneity among studies. (b) *I* ^2^: the percentage of variability due to heterogeneity. Subgroup and meta‐regression analyses were conducted to identify possible sources of variability.

Subgroup analyses explored whether specific study characteristics influenced results: (a) Participants' gender: Comparing effects in males and females; (b) Speech type: Comparing spontaneous speech, defined as unplanned spoken segments produced naturally in communicative contexts such as dialogues, interviews, or improvized presentations, versus standardized speech, the oral recitation of a pre‐specified text (Gabler et al. 2023); (c) Stress induction method: Comparing validated laboratory methods versus naturalistic stressors. Specifically, validated stressors are experimentally designed protocols with established reliability, such as the Trier Social Stress Test or the Cold Pressor Test (Allen et al. 2017; Fanninger et al. 2023), while naturalistic stressors occur in real‐world contexts, like clinical shifts, academic exams.

To assess publication bias, an inspection of the funnel plot and Egger's test were used. If significant asymmetry was found, trim‐and‐fill methods were applied to correct for bias effects. Meta‐analysis results were presented using forest plots showing individual and overall study effects. Graphs were generated using the R package ‘meta’. A sensitivity analysis was performed to test result robustness. Studies were removed one by one (‘leave‐one‐out’ method) to evaluate if any single study significantly altered the overall meta‐analysis results.

## 3 Results

### 3.1 General Aspects

**FIGURE 1:**
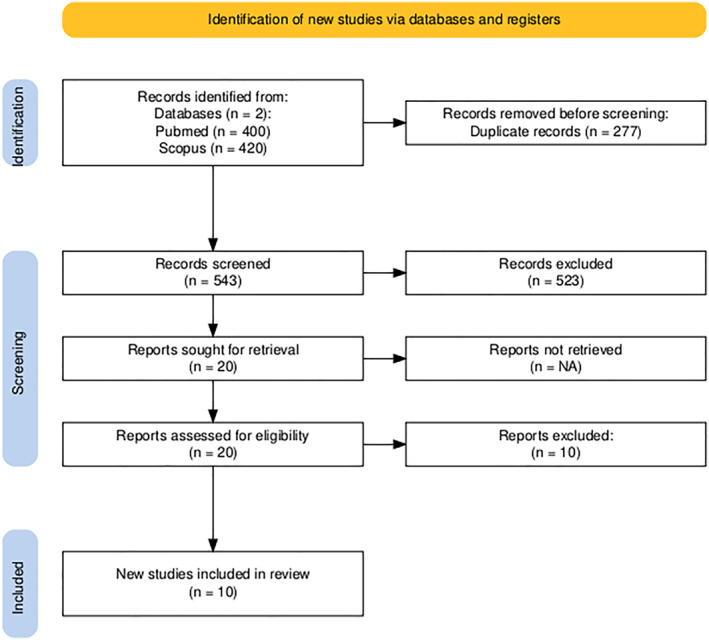
PRISMA fluxogram.

The study selection process followed PRISMA guidelines (Preferred Reporting Items for Systematic Reviews and Meta‐Analyses), as shown in Figure 1. Initially, 820 records were identified from two databases: PubMed (*n* = 400) and Scopus (*n* = 420). After removing 277 duplicates, 543 studies remained for title and abstract screening. Of these, 523 were excluded for not meeting the predefined inclusion criteria. Thus, 20 articles were selected for full‐text assessment. After a detailed review, 10 studies were excluded. In the end, a total of 10 unique studies met the eligibility criteria; however, several studies reported results for multiple independent subgroups (e.g., by gender or speech type), resulting in 15 study arms included in the meta‐analysis (Alvear et al. 2013; Giddens et al. 2010; Kappen et al. 2024; Kappen, Hoorelbeke, et al. 2022; Kappen, van der Donckt, et al. 2022; Opladen et al. 2023; Perrine and Scherer 2020; Pisanski et al. 2016; Pisanski et al. 2018; Song and Lee 2024). The studies and subgroups' data are presented in Table 1.

**TABLE 1:** Studies and subgroups data.

Study or subgroup	Speech type	Gender	Stress procedure	Method	*n*
Kappen et al. (2024)	Standardized	Female and male	Validated	MIST	66
Song and Lee (2024)	Spontaneous	Female	Naturalistic	ICU shifts	15
Song and Lee (2024)	Spontaneous	Female	Naturalistic	ICU shifts	15
Song and Lee (2024)	Spontaneous	Female	Naturalistic	ICU shifts	15
Song and Lee (2024)	Spontaneous	Female	Naturalistic	ICU shifts	15
Opladen et al. (2023)	Spontaneous	Female	Naturalistic	Body exposure	73
Kappen, van der Donckt, et al. (2022)	Standardized	Female and male	Validated	Raven's matrix	77
Kappen, Hoorelbeke, et al. (2022)	Standardized	Female and male	Validated	MIST	148
Perrine and Scherer (2020)	Standardized	Female	Validated	TSST	10
Pisanski et al. (2018)	Spontaneous	Female	Validated	TSST	47
Pisanski et al. (2018)	Spontaneous	Male	Validated	TSST	33
Pisanski et al. (2016)	Spontaneous and standardized	Female	Naturalistic	Oral academic examination	34
Alvear et al. (2013)	Standardized	Female and male	Validated	Cognitive task	14
Giddens et al. (2010)	Standardized	Male	Validated	CPT	6
Giddens et al. (2010)	Standardized	Female	Validated	CPT	6

Abbreviations: CPT, Cold Pressor Test; ICU, Intensive Care Unit; MIST, Montreal Image Stress Test; TSST, Trier Social Stress Test.

### 3.2 Demographic Data

The pooled sample included 583 participants: 53% female, 22% male; with the remainder unspecified. The participants' mean age ranged from 18.83 to 31.6 years, with a full age span between 18 and 46 years. Accordingly, most studies focused on young adults, see Table 2 for details.

**TABLE 2:** Demographic data.

Study	*n*	Age (M)	Age range	Age (SD)
Kappen et al. (2024)	66	21.29	†	2.82
Song and Lee (2024)	60	31.6	25–37	3.7
Opladen et al. (2023)	73	23.1	18–36	3.2
Kappen, van der Donckt, et al. (2022)	77	23.13	†	6.19
Kappen, Hoorelbeke, et al. (2022)	148	26.7	†	12.5
Perrine and Scherer (2020)	19	18.83	18–23	1.47
Pisanski et al. (2018)	80	†	†	†
Pisanski et al. (2016)	34	22.7	21–32	2
Alvear et al. (2013)	14	22	†	1.3
Giddens et al. (2010)	12	†	20–29	†
TOTAL	583			

*Note:* †‐ Data not provided.

### 3.3 Risk of Bias Analysis

Risk of bias in the included studies was assessed using the ROBINS‐I tool (Sterne et al. 2016) (Risk Of Bias In Nonrandomised Studies of Interventions). Of the 10 included studies, two were rated low overall risk of bias, three moderate, and five serious. The most frequent concerns involved measurement of outcomes and confounding, with additional issues in classification of interventions, selective reporting, and deviations from intended interventions; selection was generally low risk (see Table 3).

**TABLE 3:** Graphical representation of risk of bias judgements for each included study, based on the ROBINS‐I (Risk Of Bias In Nonrandomised Studies ‐ of Interventions) tool.

Study	D1	D2	D3	D4	D5	D6	D7	Overall
Kappen et al. (2024)	Low	Low	Low	Low	Low	Low	Low	Low
Song and Lee (2024)	Serious	Moderate	Moderate	Serious	Moderate	Serious	Moderate	Serious
Opladen et al. (2023)	Moderate	Moderate	Serious	Serious	Low	Serious	Moderate	Serious
Kappen, van der Donckt, et al. (2022)	Low	Low	Low	Low	Low	Low	Low	Low
Kappen, Hoorelbeke, et al. (2022)	Moderate	Low	Low	Low	Low	Low	Moderate	Moderate
Perrine and Scherer (2020)	Moderate	Moderate	Moderate	Moderate	Moderate	Serious	Moderate	Serious
Pisanski et al. (2018)	Moderate	Moderate	Low	Low	Low	Moderate	Moderate	Moderate
Pisanski et al. (2016)	Moderate	Moderate	Low	Low	Low	Moderate	Moderate	Moderate
Alvear et al. (2013)	Serious	Moderate	Moderate	Low	Low	Serious	Moderate	Serious
Giddens et al. (2010)	Serious	Serious	Moderate	Low	Moderate	Serious	Serious	Serious

### 3.4 General Meta‐Analysis Results

**FIGURE 2:**
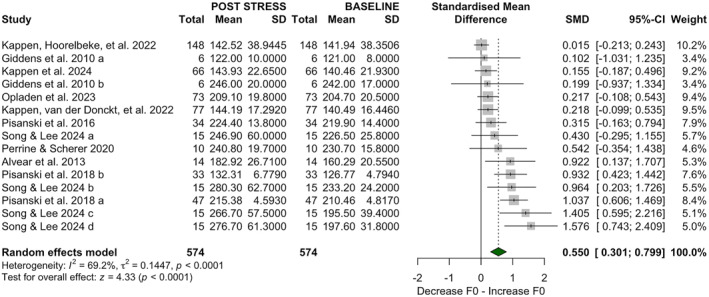
Forest plot displaying the standardized mean differences (SMD) in fundamental frequency (F0) variation after stress exposure across 15 study subgroups, derived from 10 studies, without stratification.

A random‐effects model across 15 studies (1148 observations) showed a moderate increase in F0 post‐stress (SMD = 0.55, 95% CI [0.30, 0.80], *p* < 0.001, *τ* ^2^ = 0.1447, *τ* = 0.3803, *Q* (14) = 45.48, *I* ^2^ = 69.2%), with substantial heterogeneity (see Figure 2).

#### 3.4.1 Stratified Analysis by Participants' Gender

**FIGURE 3:**
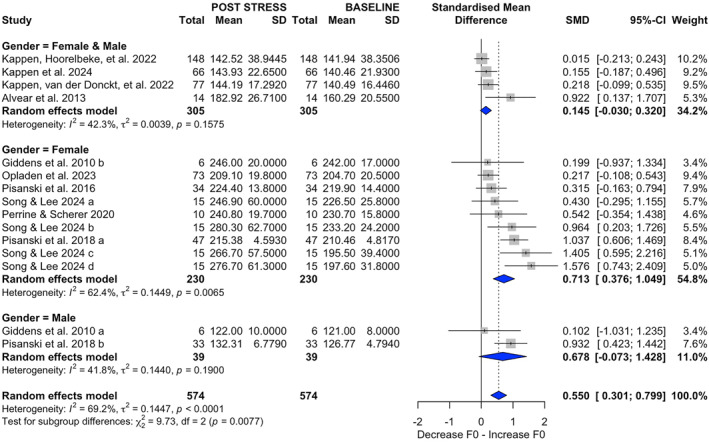
Forest plot displaying the standardized mean differences (SMD) in fundamental frequency (F0) variation after stress exposure, stratified by gender. In the first plot (top), four subgroups with mixed gender participants are presented. In the second plot (middle), nine subgroups with female participants are presented, In the third plot (bottom), two subgroups with male participants are presented.

In the gender analysis (Figure 3), nine female‐only studies (460 observations) produced a moderate, significant effect (SMD = 0.7128; 95% CI [0.3763, 1.0492]; *p* < 0.0001) with substantial heterogeneity (*τ* ^2^ = 0.1449; *τ* = 0.3806; *Q* = 21.27; *I* ^2^ = 62.4%). Two male‐only studies (78 observations) indicated SMD = 0.6775 but did not reach significance (95% CI [−0.0732, 1.4282]; *p* = 0.078; *τ* ^2^ = 0.1440; *τ* = 0.3795; *Q* = 1.72; *I* ^2^ = 41.8%). Four mixed‐gender studies (610 observations) showed a smaller, nonsignificant estimate (SMD = 0.1449; 95% CI [−0.0298, 0.3196]; *p* = 0.102; *τ* ^2^ = 0.0039; *τ* = 0.0627; *Q* = 5.20; *I* ^2^ = 42.3%). The subgroup comparison was significant (*Q* = 9.73; *p* = 0.0077).

#### 3.4.2 Stratified Analysis by Speech Type

**FIGURE 4:**
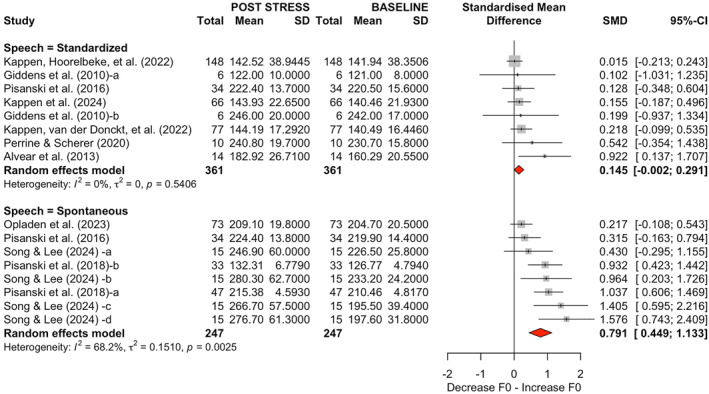
Forest plot displaying the standardized mean differences (SMD) in fundamental frequency (F0) variation after stress exposure, stratified by speech type. In the first plot (top), eight subgroups involve standardized speech, while in the second plot (bottom), eight subgroups involve spontaneous speech. Note that the study by Pisanski et al. (2016) appears in both plots, as it included both types of experiments.

In the speech‐type analysis (Figure 4), eight studies using spontaneous speech (494 observations) showed a large, statistically significant effect (SMD = 0.7911; 95% CI [0.4492, 1.1331]; *p* < 0.0001) with moderate heterogeneity (*τ* ^2^ = 0.1510; *τ* = 0.3886; *Q* = 22.04; *I* ^2^ = 68.2%). By contrast, eight studies employing standardized speech (722 observations) produced a small, nonsignificant estimate (SMD = 0.1449; 95% CI [−0.0016, 0.2913]; *p* = 0.052) and low heterogeneity (*τ* ^2^ = 0; *τ* = 0; *Q* = 5.99; *I* ^2^ = 0.0%).

#### 3.4.3 Stratified Analysis by Stress Induction Method

**FIGURE 5:**
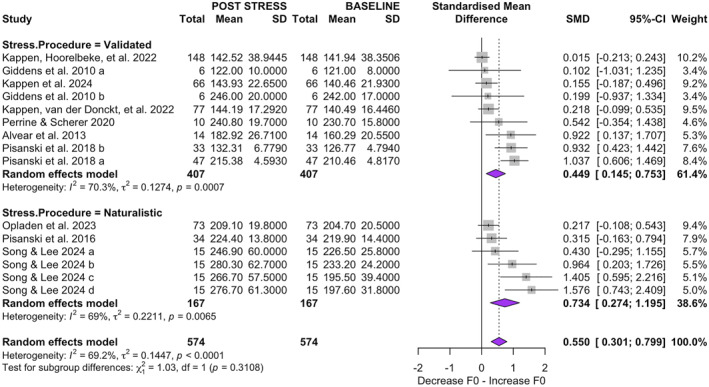
Forest plot displaying the standardized mean differences (SMD) in fundamental frequency (F0) variation after stress exposure, stratified by stress induction procedure. In the first plot (top), nine subgroups involve validated stress procedures, while in the second plot (bottom), six subgroups involve naturalistic stress procedures.

In the subgroup analysis of stress induction methods (Figure 5), six studies employing naturalistic stress scenarios (334 observations) yielded a large, statistically significant effect (SMD = 0.7344; 95% CI [0.2738, 1.1951]; *p* = 0.002) with moderate heterogeneity (*τ* ^2^ = 0.2211; *τ* = 0.4702; *Q* = 16.14; *I* ^2^ = 69.0%). By contrast, nine studies using validated laboratory procedures (814 observations) produced a smaller but still significant estimate (SMD = 0.4490; 95% CI [0.1449, 0.7532]; *p* = 0.004) alongside high heterogeneity (*τ* ^2^ = 0.1274; *τ* = 0.3569; *Q* = 26.93; *I* ^2^ = 70.3%). The between‐group comparison was not significant (*Q* = 1.03; *p* = 0.3108).

#### 3.4.4 Publication Bias

Evidence of asymmetry was indicated by Egger's test (*p* = 0.011). Applying the trim‐and‐fill method, seven studies were imputed, adjusting the total number to 22, the corrected effect size was (SMD = 0.17, 95% CI [−0.15, 0.49], *p* = 0.292) and was no longer statistically significant. These findings suggest that the original effect (SMD = 0.5504) may have been inflated due to publication bias. Heterogeneity remained high after correction (*I* ^2^ = 81.2%). A leave‐one‐out analysis was conducted to evaluate the influence of individual studies on the overall meta‐analytic effect. The SMDs ranged from 0.487 to 0.606, with all 95% confidence intervals excluding zero and all *p*‐values remaining statistically significant (*p* < 0.001).

## 4 Discussion

This review shows that stress significantly affects the fundamental frequency (F0) of voice. The overall analysis showed that acute stress significantly influences F0, with a moderate effect (SMD = 0.55, 95% CI [0.30, 0.80], *p* < 0.001). This moderate F0 increase aligns with physiological models suggesting that stress‐induced laryngeal tension stiffens vocal cords, raising vibration frequency (Van Puyvelde et al. 2018; Zhou et al. 2001).

Acute stress triggers two primary physiological systems: the sympathetic nervous system (SNS) and the hypothalamic–pituitary–adrenal (HPA) axis, producing autonomic and endocrine changes relevant to vocal control (Agorastos and Chrousos 2022). In central networks, the anterior cingulate cortex integrates arousal regulation with vocal motor activity and connects with the vagus, highlighting respiration as a link between stress and voice. Through the vagus, the superior laryngeal branch innervates the cricothyroid muscle, while the recurrent laryngeal branch supplies the remaining intrinsic laryngeal muscles, routes by which autonomic drive alters stiffness and adduction that influence F0 (Câmara and Griessenauer 2015; Van Puyvelde et al. 2018). Experiments have shown that intrinsic laryngeal muscles often exhibit rapid increased activation under acute stress (Helou et al. 2020). Greater tension in the vocal folds causes them to vibrate faster, producing a higher frequency sound (Dichter et al. 2018).Taken together, these autonomic, central, and laryngeal pathways may provide a plausible physiological basis for the observed F0 elevations during acute stress.

Beyond these physiological mechanisms, the meta‐analysis also revealed considerable heterogeneity across studies (*I* ^2^ = 69.2%). Subgroup analyses were conducted based on theoretical considerations that study and participant characteristics may modulate the stress‐voice coupling. Gender may act as a moderator of the stress effect on F0. Fundamental frequency characteristics are one of the strongest cues for gender perception by listeners (Skuk and Schweinberger 2014). The moderation may also be linked to hormonal differences, such as testosterone levels, which are inversely related to F0 in men (Evans et al. 2008), as well as with differences in the structure of the larynx and vocal folds, where androgen and oestrogen receptors are present (Voelter et al. 2008). Accordingly, the gender subgroup analysis revealed a significant effect in women (SMD = 0.71, 95% CI [0.38, 1.05], *p* < 0.001, *I* ^2^ = 62.4%), but were not significant in men (SMD = 0.6775; 95% CI [–0.0732, 1.4282]; *p* = 0.078) or in mixed samples, subgroup analysis revealed significant differences (*Q* = 9.73; *p* = 0.0077). Sample composition favoured mixed‐gender cohorts and included few male‐only studies. This distribution reduces power for male‐specific estimates and widens their confidence intervals. Taken together, these features suggest that clearer insights could come from sex‐specific, adequately powered studies with complete gender reporting, which could help determine whether F0 can serve as a broadly reliable indicator of stress.

In addition to gender, characteristics of the speech sample and stressor type also influenced outcomes. Spontaneous speech showed a strong effect (SMD = 0.7911; 95% CI = [0.4492; 1.1331]), while standardized speech did not show significance (SMD = 0.1449; 95% CI = [−0.0016; 0.2913]). Although read and spontaneous methods share similar acoustic features, in spontaneous speech, the variability of the F0 plays a more prominent role than in read speech (Lee and Kreiman 2022).

Regarding stressor type, naturalistic scenarios had a stronger effect (SMD = 0.7344; 95% CI = [0.2738; 1.1951]) than validated ones (SMD = 0.4490; 95% CI = [0.1449; 0.7532]). Previous studies comparing laboratory protocols, such as the Trier Social Stress Test (TSST), with real‐world stress situations, like oral examinations or public exposure, show that while both can elicit similar physiological responses, including increases in salivary cortisol and heart rate, important differences remain in the dynamics and intensity of the affective response. Notably, the impact on negative affect tends to be more pronounced in naturalistic settings (Henze et al. 2017). Furthermore, prior experience with the stressor (e.g., actors accustomed to public speaking) can lead to cross‐adaptation, resulting in attenuated physiological responses in both settings, particularly at the level of heart rate (Jezova et al. 2016). Also, individuals may adapt more quickly to laboratory stressors. If an individual receives stressor pre‐exposure and information about the test, they exhibit reduced psychological and physiological stress responses, suggesting that familiarity can attenuate stress effects (Cohen 1994). Naturalistic stressors, on the other hand, are often new and unexpected, which may lead to a stronger initial response. Rather than privileging one paradigm over the other, it is more important to recognise that laboratory and naturalistic contexts offer complementary settings for analysing voice–stress coupling; importantly, the feasibility of naturalistic capture supports translational prospects in clinical, occupational, and everyday use.

Despite these insights, important methodological issues remain. Studies standardisation is lacking, including stress induction methods, speech types, and voice recording protocols. This methodological diversity complicates the comparison and synthesis of results across studies and may contribute to heterogeneity. While validated procedures such as the Trier Social Stress Test (TSST) (Allen et al. 2017) and the Montreal Imaging Stress Task (MIST) (Dedovic et al. 2005) have been widely adopted, even studies using the same protocols differ in exposure duration, speech tasks, and timing of data collection. Factors such as microphone type, recording environment, software, and time of data collection could not be analysed but may have influenced the results. Technical aspects like room reverberation, microphone characteristics, and software processing can introduce additional errors. For instance, studies have shown that measures such as shimmer and HNR become unstable when reverberation time exceeds one second, even in clear vocal styles (Yousef and Hunter 2024), highlighting the importance of recording conditions as essential for ensuring comparability and reliability across studies.

However, a key consideration in this study is the publication bias, which substantially qualifies the inference from the primary meta‐analysis. Funnel‐plot asymmetry and Egger's test (*p* = 0.011) indicated small‐study effects; after application of the trim‐and‐fill procedure, the pooled estimate was attenuated and no longer statistically significant (SMD = 0.1710; 95% CI [−0.1472, 0.4891]; *p* = 0.2923). Accordingly, the F0 finding should be regarded as preliminary, and stand‐alone biomarker claims are not warranted at this stage. The included studies also showed substantial methodological heterogeneity (*I* ^2^ = 69.2%), suggesting context‐dependent effects. Although leave‐one‐out analyses indicate that no observed study disproportionately drives the unadjusted result, this sensitivity check cannot address bias arising from unobserved works, and heterogeneity remained high after adjustment (*I* ^2^ = 81.2%). Risk‐of‐bias ratings indicated a predominance of moderate and serious overall risk (two low, three moderate, five serious), with recurrent concerns in confounding and outcome measurement. It suggests that some effects on F0 may be overestimated and contribute to between‐study heterogeneity. These considerations support a cautious interpretation and underscore the need for preregistered, adequately powered studies with standardized acoustic assessment and routine reporting of null findings to clarify the magnitude and reliability of F0‐based stress inferences.

These results suggest that the evaluation of F0 variation may be useful in detecting and monitoring stress across contexts. Studies have already used F0 variation as an indicator of psychological stress, such as the study by Hall (Hall et al. 2021), which used it as a marker of stress during surgical simulation. An important consideration is that F0 elevations are not exclusive to stress. F0 typically increases in response to any intensification of physiological arousal, including emotions such as excitement, anger, or nonstructural fear (Giddens et al. 2013). Pitch often rises under stress; however, this effect is not universal and varies with the emotional state and the individual's baseline autonomic tone. For example, voices expressing joy or irritation also present elevated pitch (Kamiloğlu et al. 2020). Arousal may also present as hyperfunctional laryngeal postures (e.g., supraglottic compression, elevated laryngeal position), which stiffen the vibratory system and are associated with higher F0 (Ferrán 2024; Hillman et al. 2020). Therefore, caution is necessary when attributing an F0 increase exclusively to stress; it may reflect a general state of emotional arousal (Bänziger and Scherer 2005). Distinguishing negative stress from other high‐arousal states based on voice alone will likely require integrating other signals or contextual information (Giddens et al. 2013). By comparison, noninvasive physiological markers such as heart rate variability (HRV) has already been identified as a possible stress indicator and can be recorded with portable devices (Presby et al. 2023; Tomes et al. 2025). It has even been evaluated for estimating workload and allostatic load in tactical police groups, showing good sensitivity (Tomes et al. 2025), and its application is being considered in several mental health conditions and anxiety (Heiss et al. 2021; Tomasi et al. 2024). While voice acoustic parameters, particularly F0, have shown promise as stress indicators, their validation and clinical applicability remain in earlier stages compared to well‐established measures like heart rate variability (HRV). Further research is necessary to determine whether voice analysis can complement or approximate the diagnostic utility of HRV in diverse settings. While HRV is more established, voice analysis offers unique practical advantages; acoustic features are acquired passively and without physical contact, potentially reducing white coat effects (Townsend and Cohen 2024). Some studies explore their use as a tool for day‐long tracking of stress fluctuations (Langer et al. 2022), and their real‐time monitoring may serve as an alert for increasing stress levels throughout the day. In low‐resource environments, acoustic markers offer a practical advantage because voice recordings can be easily captured and analysed. Today, mobile phones, which are widely available and capable of recording sound, make this even more feasible. According to the 2023 ICT Households Survey, 93% of Brazilians aged 10 and older use a mobile phone, and 86% of individuals in social classes D/E already have access to one (Núcleo de Informação e Coordenação do Ponto BR 2024).

Previous studies have linked vocal parameters to depressive symptoms and emotional stress, like depression, anxiety, bipolar disorder, and schizophrenia (Albuquerque et al. 2021; Briganti and Lechien 2025; Low et al. 2020; Menne et al. 2024; Parola et al. 2023; Takano et al. 2023). The use of F0 to assess emotional stress in survivors of head and neck cancer was evaluated, noting a relationship between acoustic changes and stress, and a possible use of this approach in clinical settings as a practical resource (Kandsberger et al. 2016). Integration with artificial intelligence is also promising, with algorithms identifying multiple vocal patterns linked to psychological states, symptoms, and risks, such as the suicide risk (Min et al. 2023).

Despite promising results, this study has limitations. Methodological diversity among studies makes generalisation difficult, underscoring the need to standardise stress induction methods and acoustic data collection. For clinical utility, a vocal biomarker should present adequate sensitivity and specificity to reliably distinguish stressed from nonstressed states. Evidence to date is insufficient to establish these parameters for F0. Environmental factors, such as background noise, microphone quality, and recording distance, may significantly affect measurement reliability and should be controlled in experimental and applied settings. Furthermore, the minimum detectable change in F0 that represents a meaningful stress response remains to be determined. Addressing these factors will be essential to translate voice analysis into robust clinical and occupational tools.

Moreover, most studies focused solely on F0 as the main parameter. Although other parameters like shimmer, jitter, and HNR appeared in some studies, they were less frequently reported. Future research should include additional acoustic markers to broaden the understanding of stress effects on voice.

Recommendations for future studies include: (A) The adoption of standardized protocols for stress induction and acoustic data collection, encompassing both standardized and spontaneous speech samples, as well as gender‐stratified cohorts with with sufficient male representation; (B) Investigation of other vocal markers such as jitter, shimmer, and formants for broader analysis; (C) Greater population diversity, with larger and more varied samples from different social and cultural contexts; (D) Longitudinal studies to track participants over time for deeper insights into stress and voice dynamics; (E) Application of artificial intelligence to improve joint analysis of acoustic parameters and their correlation with physiological stress markers.

Thus, the data from this review support the use of acoustic markers, specifically the fundamental frequency, as a possible tool in stress evaluation, contributing to scientific research and clinical practice, like automated detection of stress, computing indirect allostatic load on workers, and live evaluation of stress burden of multiple medical procedures.

## 5 Conclusion

This systematic review and meta‐analysis indicates that acute stress elevates vocal F0, with significant effects in female subgroups and in spontaneous speech. Given the heterogeneity and publication bias, results should be interpreted cautiously. Accordingly, F0 as a noninvasive candidate biomarker requires validation in large, prospective studies using standardized protocols and representative, gender‐stratified cohorts.

## Ethics Statement

This study used publicly available data and did not require approval from an ethics committee.

## Consent

The research used only publicly available and anonymised data; no patient consent was needed.

## Conflicts of Interest

The authors declare no conflicts of interest.

## Systematic Review Registration

This systematic review was registered in the PROSPERO database (registration number: CRD4202347652).

## Supporting information


Supporting Information S1

## Data Availability

The data that supports the findings of this study are available in the supplementary material of this article.
