# Supplementary material for: The Fundamental Frequency of Voice as a Potential Stress Biomarker: A Systematic Review and Meta–Analysis
Source: Stress Health. 2025 Oct 16;41(5):e70112. doi: 10.1002/smi.70112 (PMC12531429; doi:10.1002/smi.70112)
Supplement: Supplementary file 1 — Supporting Information S1 [file SMI-41-e70112-s001.docx]

**The Fundamental Frequency of Voice as a Potential Stress Biomarker: a literature review and metanalysis.**

# Supplementary Material

## Eligibility Criteria

The following databases were reviewed: PubMed/Medline and Scopus, for articles published from 2010 to September 2024. The search terms used in each database are displayed in supplementary material:

**PubMed/Medline**:
"stress, psychological"[MeSH Terms] OR "anxiety"[MeSH Terms] OR "stress"[All Fields] OR "stressed"[All Fields] OR "stresses"[All Fields] OR "stressful"[All Fields] OR "stressfulness"[All Fields] OR "stressing"[All Fields] OR "stress detection"[All Fields] AND "speech acoustics"[MeSH Terms] OR "acoustic features"[All Fields] OR "Harmonics-to-noise ratio"[All Fields] OR "jitter"[All Fields] OR "jittered"[All Fields] OR "jittering"[All Fields] OR "jitters"[All Fields] OR "shimmer"[All Fields] OR "shimmers"[All Fields] OR "fundamental frequency"[All Fields].

**Scopus**:
"acoustic features" OR "speech acoustics" OR "Harmonics‐to‐noise ratio" OR "jitter" OR "Shimmer" OR "fundamental frequency” AND “psychological stress” OR “stress” OR “anxiety” OR "stress detection".

## Demographic data

| **Study** | **n** | **Age (mean)** | **Interval** | **Age (SD)** | **N Female** | **Female Age(mean)** | **Female interval** | **Female Age (SD)** | **N Male** | **Male Age (mean)** | **Male interval** | **Male Age (SD)** |
| --- | --- | --- | --- | --- | --- | --- | --- | --- | --- | --- | --- | --- |
| Kappen M  2024(Kappen et al., 2024) | 66 | 21,29 |  | 2,82 | 13 |  |  |  | 53 |  |  |  |
| Song Z 2024(Song & Lee, 2024) | 60 | 31,6 | 25-37 | 3,7 | 60 | 31,6 | 25-37 | 3,7 |  |  |  |  |
| Opladen  2023(Opladen et al., 2023) | 73 | 23,1 | 18-36 | 3,2 | 73 | 23,1 | 18-36 | 3,2 |  |  |  |  |
| Kappen M 2022-a(Kappen, van der Donckt, et al., 2022) | 77 | 23,13 |  | 6,19 | 50 |  |  |  | 27 |  |  |  |
| Kappen M  2022-b(Kappen, Hoorelbeke, et al., 2022) | 148 | 26,7 |  | 12,5 |  |  |  |  |  |  |  |  |
| Perrine BL 2020(Perrine & Scherer, 2020) | 19 | 18,83 | 18-23 | 1,47 | 19 | 18,83 | 18-23 | 1,47 |  |  |  |  |
| Pisanski K 2018(Pisanski et al., 2018) | 80 |  |  |  | 47 | 24,8 | 19-41 | 3,2 | 33 | 26,6 | 21-46 | 4 |
| Pisanski K 2016(Pisanski et al., 2016) | 34 | 22,7 | 21-32 | 2 | 34 | 22,7 | 21.32 | 2 |  |  |  |  |
| Alvear RM 2013(Alvear et al., 2013) | 14 | 22 |  | 1,3 | 7 |  |  |  | 7 |  |  |  |
| Giddens CL  2010(Giddens et al., 2010) | 12 |  | 20-29 |  | 6 |  |  |  | 6 |  |  |  |
| TOTAL | 583 |  |  |  | 309 |  |  |  | 126 |  |  |  |

Table 1. Demographic data

## Included reports

Table 2 Included Reports

| **Title** | **Year** | **Journal** | **ISSN** | **Authors** | **Abstract** | **doi** |
| --- | --- | --- | --- | --- | --- | --- |
| Acoustic and prosodic speech features reflect physiological stress but not isolated negative affect: a multi-paradigm study on psychosocial stressors. | 2024 | Scientific reports | 2045-2322 (Electronic) | Kappen M and Vanhollebeke G and Van Der Donckt J and Van Hoecke S and Vanderhasselt MA(Kappen et al., 2024) | Heterogeneity in speech under stress has been a recurring issue in stress research, potentially due to varied stress induction paradigms. This study investigated speech features in semi-guided speech following two distinct psychosocial stress paradigms (Cyberball and MIST) and their respective control conditions. Only negative affect increased during Cyberball, while self-reported stress, skin conductance response rate, and negative affect increased during MIST. Fundamental frequency (F0), speech rate, and jitter significantly changed during MIST, but not Cyberball; HNR and shimmer showed no expected changes. The results indicate that observed speech features are robust in semi-guided speech and sensitive to stressors eliciting additional physiological stress responses, not solely decreases in negative affect. These differences between stressors may explain literature heterogeneity. Our findings support the potential of speech as a stress level biomarker, especially when stress elicits physiological reactions, similar to other biomarkers. This highlights its promise as a tool for measuring stress in everyday settings, considering its affordability, non-intrusiveness, and ease of collection. Future research should test these results' robustness and specificity in naturalistic settings, such as freely spoken speech and noisy environments while exploring and validating a broader range of informative speech features in the context of stress. | 10.1038/s41598-024-55550-3 |
| Voice use of nurses working in the intensive care unit during the COVID-19 pandemic. | 2024 | Intensive & critical care nursing | 1532-4036 (Electronic) | Song Z and Lee PJ(Song & Lee, 2024) | OBJECTIVE: This study aimed to investigate the voice use of nurses working in intensive care units (ICUs) and their perception of acoustic environments. SETTING AND SAMPLE: The research was conducted in four different hospitals in China during the COVID-19 pandemic. A total of 60 ICU nurses were recruited for their voice use monitoring and 100 nurses participated in the survey. RESEARCH METHODOLOGY: Firstly, voice-related parameters such as voice level (SPL, dB), fundamental frequency (F0, Hz), and voicing time percentage (Dt, %) were measured using a vocal monitor. To collect data, a non-invasive accelerometer was attached to the participants' necks during their working hours. Secondly, the perception of the ICU acoustic environment was assessed using semantic differential. RESULTS: The results showed that nurses spoke approximately 0.9-4 dB louder to patients and colleagues in ICUs compared to quiet rooms, and their fundamental frequency (F0) significantly increased during work. The voice levels of nurses were influenced by background noise levels, with a significant correlation coefficient of 0.44 (p < 0.01). Furthermore, the background noise levels ranged from 58.1 to 73.9 dBA, exceeding the guideline values set by the World Health Organisation (WHO). The semantic differential analysis identified 'Stress' and 'Irritation' as the two main components, indicating the prevalence of negative experiences within ICUs. IMPLICATIONS FOR CLINICAL PRACTICE: This study highlights the potential risk of voice disorders among ICU nurses. The findings also underscore the importance of implementing strategies to reduce noise levels in ICUs to reduce voice disorders among nurses. | 10.1016/j.iccn.2023.103620 |
| Body exposure and vocal analysis: validation of fundamental frequency as a correlate of emotional arousal and valence. | 2023 | Frontiers in psychiatry | 1664-0640 (Print) | Opladen V and Tanck JA and Baur J and Hartmann AS and Svaldi J and Vocks S(Opladen et al., 2023) | INTRODUCTION: Vocal analysis of fundamental frequency (f0) represents a suitable index to assess emotional activation. However, although f0 has often been used as an indicator of emotional arousal and different affective states, its psychometric properties are unclear. Specifically, there is uncertainty regarding the validity of the indices of f0(mean) and f0(variabilitymeasures) (f0(dispersion), f0(range), and f0(SD)) and whether higher or lower f0 indices are associated with higher arousal in stressful situations. The present study therefore aimed to validate f0 as a marker of vocally encoded emotional arousal, valence, and body-related distress during body exposure as a psychological stressor. METHODS: N = 73 female participants first underwent a 3-min, non-activating neutral reference condition, followed by a 7-min activating body exposure condition. Participants completed questionnaires on affect (i.e., arousal, valence, body-related distress), and their voice data and heart rate (HR) were recorded continuously. Vocal analyses were performed using Praat, a program for extracting paralinguistic measures from spoken audio. RESULTS: The results revealed no effects for f0 and state body dissatisfaction or general affect. F0(mean) correlated positively with self-reported arousal and negatively with valence, but was not correlated with HR(mean/maximum). No correlations with any measure were found for any f0(variabililtymeasures). DISCUSSION: Given the promising findings regarding f0(mean) for arousal and valence and the inconclusive findings regarding f0 as a marker of general affect and body-related distress, it may be assumed that f0(mean) represents a valid global marker of emotional arousal and valence rather than of concrete body-related distress. In view of the present findings regarding the validity of f0, it may be suggested that f0(mean), but not f0(variabilitymeasures), can be used to assess emotional arousal and valence in addition to self-report measures, which is less intrusive than conventional psychophysiological measures. | 10.3389/fpsyt.2023.1087548 |
| Acoustic speech features in social comparison: how stress impacts the way you sound. | 2022 | Scientific reports | 2045-2322 (Electronic) | Kappen M and van der Donckt J and Vanhollebeke G and Allaert J and Degraeve V and Madhu N and Van Hoecke S and Vanderhasselt MA(Kappen, Hoorelbeke, et al., 2022) | The use of speech as a digital biomarker to detect stress levels is increasingly gaining attention. Yet, heterogeneous effects of stress on specific acoustic speech features have been observed, possibly due to previous studies' use of different stress labels/categories and the lack of solid stress induction paradigms or validation of experienced stress. Here, we deployed a controlled, within-subject psychosocial stress induction experiment in which participants received both neutral (control condition) and negative (negative condition) comparative feedback after solving a challenging cognitive task. This study is the first to use a (non-actor) within-participant design that verifies a successful stress induction using both self-report (i.e., decreased reported valence) and physiological measures (i.e., increased heart rate acceleration using event-related cardiac responses during feedback exposure). Analyses of acoustic speech features showed a significant increase in Fundamental Frequency (F0) and Harmonics-to-Noise Ratio (HNR), and a significant decrease in shimmer during the negative feedback condition. Our results using read-out-loud speech comply with earlier research, yet we are the first to validate these results in a well-controlled but ecologically-valid setting to guarantee the generalization of our findings to real-life settings. Further research should aim to replicate these results in a free speech setting to test the robustness of our findings for real-world settings and should include semantics to also take into account what you say and not only how you say it. | 10.1038/s41598-022-26375-9 |
| Speech as an indicator for psychosocial stress: A network analytic approach. | 2022 | Behavior research methods | 1554-3528 (Electronic) | Kappen M and Hoorelbeke K and Madhu N and Demuynck K and Vanderhasselt MA(Kappen, Hoorelbeke, et al., 2022) | Recently, the possibilities of detecting psychosocial stress from speech have been discussed. Yet, there are mixed effects and a current lack of clarity in relations and directions for parameters derived from stressed speech. The aim of the current study is - in a controlled psychosocial stress induction experiment - to apply network modeling to (1) look into the unique associations between specific speech parameters, comparing speech networks containing fundamental frequency (F0), jitter, mean voiced segment length, and Harmonics-to-Noise Ratio (HNR) pre- and post-stress induction, and (2) examine how changes pre- versus post-stress induction (i.e., change network) in each of the parameters are related to changes in self-reported negative affect. Results show that the network of speech parameters is similar after versus before the stress induction, with a central role of HNR, which shows that the complex interplay and unique associations between each of the used speech parameters is not impacted by psychosocial stress (aim 1). Moreover, we found a change network (consisting of pre-post stress difference values) with changes in jitter being positively related to changes in self-reported negative affect (aim 2). These findings illustrate - for the first time in a well-controlled but ecologically valid setting - the complex relations between different speech parameters in the context of psychosocial stress. Longitudinal and experimental studies are required to further investigate these relationships and to test whether the identified paths in the networks are indicative of causal relationships. | 10.3758/s13428-021-01670-x |
| Aerodynamic and Acoustic Voice Measures Before and After an Acute Public Speaking Stressor. | 2020 | Journal of speech, language, and hearing research : JSLHR | 1558-9102 (Electronic) | Perrine BL and Scherer RC(Perrine & Scherer, 2020) | Purpose The goal of this study was to determine if differences in stress system activation lead to changes in speaking fundamental frequency, average oral airflow, and estimated subglottal pressure before and after an acute, psychosocial stressor. Method Eighteen vocally healthy adult females experienced the Trier Social Stress Test (TSST) to activate the hypothalamic-pituitary-adrenal axis. The TSST includes public speaking and performing mental arithmetic in front of an audience. At seven time points, three before the stressor and four after the stressor, the participants produced /pa/ repetitions, read the Rainbow Passage, and provided a saliva sample. Measures included (a) salivary cortisol level, (b) oral airflow, (c) estimated subglottal pressure, and (d) speaking fundamental frequency from the second sentence of the Rainbow Passage. Results Ten of the 18 participants experienced a hypothalamic-pituitary-adrenal axis response to stress as indicated by a 2.5-nmol/L increase in salivary cortisol from before the TSST to after the TSST. Those who experienced a response to stress had a significantly higher speaking fundamental frequency before and immediately after the stressor than later after the stressor. No other variable varied significantly due to the stressor. Conclusions This study suggests that the idiosyncratic and inconsistent voice changes reported in the literature may be explained by differences in stress system activation. In addition, laryngeal aerodynamic measures appear resilient to changes due to acute stress. Further work is needed to examine the influence of other stress systems and if these findings hold for dysphonic individuals. | 10.1044/2020_JSLHR-19-00252 |
| Multimodal stress detection: Testing for covariation in vocal, hormonal and physiological responses to Trier Social Stress Test. | 2018 | Hormones and behavior | 1095-6867 (Electronic) | Pisanski K and Kobylarek A and Jakubowska L and Nowak J and Walter A and Błaszczyński K and Kasprzyk M and Łysenko K and Sukiennik I and Piątek K and Frackowiak T and Sorokowski P(Pisanski et al., 2018) | Examining the effects of acute stress across multiple modalities (behavioral, physiological, and endocrinological) can increase our understanding of the interplay among stress systems, and may improve the efficacy of stress detection. A multimodal approach also allows for verification of the biological stress response, which can vary between individuals due to myriad internal and external factors, thus allowing for reliable interpretation of behavioral markers of stress. Here, controlling for variables known to affect the magnitude of the stress response, we utilized the Trier Social Stress Test (TSST) to elicit an acute stress response in 80 healthy adult men and women. The TSST involves an interview-style oral presentation and critical social evaluation, and is highly effective in inducing psychosocial stress. Participants completed the study in individual 2 h sessions, during which we collected voice, polygraph and salivary hormone measures in baseline, stress, and relaxation phases. Our results show sizeable systematic increases in voice pitch (mean, minimum and variation in fundamental frequency, F0), hormone levels (cortisol) and decreases in skin temperature and hand movement during psychosocial stress, with striking similarities between men and women. However, cortisol and skin temperature only weakly predicted changes in voice pitch during stress, in either women or men, respectively. Thus, while our results provide compelling evidence that psychosocial stress manifests itself behaviorally by increasing voice pitch and its variability alongside simultaneous activation of physiological and endocrinological stress systems, our results also highlight a relatively weak degree of intra-individual 'response coherence' across these stress systems, with dissociations among different stress measures related most strongly to sex. | 10.1016/j.yhbeh.2018.08.014 |
| Individual differences in cortisol stress response predict increases in voice pitch during exam stress. | 2016 | Physiology & behavior | 1873-507X (Electronic) | Pisanski K and Nowak J and Sorokowski P(Pisanski et al., 2016) | Despite a long history of empirical research, the potential vocal markers of stress remain unclear. Previous studies examining speech under stress most consistently report an increase in voice pitch (the acoustic correlate of fundamental frequency, F0), however numerous studies have failed to replicate this finding. In the present study we tested the prediction that these inconsistencies are tied to variation in the severity of the stress response, wherein voice changes may be observed predominantly among individuals who show a cortisol stress response (i.e., an increase in free cortisol levels) above a critical threshold. Voice recordings and saliva samples were collected from university psychology students at baseline and again immediately prior to an oral examination. Voice recordings included both read and spontaneous speech, from which we measured mean, minimum, maximum, and the standard deviation in F0. We observed an increase in mean and minimum F0 under stress in both read and spontaneous speech, whereas maximum F0 and its standard deviation showed no systematic changes under stress. Our results confirmed that free cortisol levels increased by an average of 74% (ranging from 0 to 270%) under stress. Critically, increases in cortisol concentrations significantly predicted increases in mean F0 under stress for both speech types, but did not predict variation in F0 at baseline. On average, stress-induced increases in voice pitch occurred only when free cortisol levels more than doubled their baseline concentrations. Our results suggest that researchers examining speech under stress should control for individual differences in the magnitude of the stress response. | 10.1016/j.physbeh.2016.05.018 |
| Interactions between voice fundamental frequency and cardiovascular parameters. Preliminary results and physiological mechanisms. | 2013 | Logopedics, phoniatrics, vocology | 1651-2022 (Electronic) | Alvear RM and Barón-López FJ and Alguacil MD and Dawid-Milner MS(Alvear et al., 2013) | OBJECTIVES: To determine heart rate influence on voice fundamental frequency under stress conditions. METHODS: In 14 healthy volunteers, heart rate and blood pressure variables were analyzed during three classical autonomic tasks. Sustained voice samples were obtained to analyze F0. RESULTS: Cold pressure test increased mean blood pressure, without effect on heart rate; isometric and mental tasks increased heart rate and blood pressure. Voice F0 was only affected by mental and cold ice tasks; it significantly correlated with the heart rate that occurred before and during every vocal emission. DISCUSSION: Cardiovascular changes showed that subjects were significantly stressed during autonomic tasks. Heartbeat variations had a regular and significant influence on phonatory frequency, and this effect occurred during baseline and stress conditions. | 10.3109/14015439.2012.696140 |
| Beta-adrenergic blockade and voice: a double-blind, placebo-controlled trial. | 2010 | Journal of voice : official journal of the Voice Foundation | 1873-4588 (Electronic) | Giddens CL and Barron KW and Clark KF and Warde WD (Giddens et al., 2010) | This study investigated the effects of laboratory-induced stress and beta-adrenergic blockade on acoustic and aerodynamic voice measures. In a double-blind, placebo-controlled trial, 12 participants, six males and six females, underwent cold pressor-induced sympathetic activation followed by placebo or treatment with 40 mg propranolol. Aerodynamic and acoustic parameters of voice were collected at baseline, during cold pressor and after treatment with propranolol or placebo. Fundamental frequency, jitter, shimmer, maximum airflow declination rate, voice onset time, speaking rate, and subglottal pressure were measured at baseline, during cold pressor-induced stress, and after treatment with propranolol or placebo. Cardiovascular measures served as indicators of sympathetic nervous system (SNS) activation by cold pressor and antagonism by propranolol, and were collected during all conditions. Cold pressor appeared to adequately agonize the SNS as indicated by significant increases in resting systolic and diastolic blood pressure and heart rate. Propranolol appeared to adequately antagonize the SNS for the participants. Jitter ratio demonstrated a statistically significant increase in the participants treated with propranolol. Speaking rate demonstrated a small but significant increase in the placebo control group during cold pressor. Gender differences were observed in a few measures. Cold pressor adequately agonized and propranolol adequately antagonized the SNS. No statistically significant differences across subjects were observed in the voice parameters during cold pressor-induced stress before treatment. Jitter ratio increased significantly during propranolol treatment and cold pressor. Speaking rate demonstrated a statistically significant increase during cold pressor in the placebo control group. Gender differences were observed, but were few. | 10.1016/j.jvoice.2008.12.002 |

Table 3 Excluded Reports

| **Title** | **Year** | **Journal** | **Authors** |  |
| --- | --- | --- | --- | --- |
| Parenting stress in parents with and without a mental illness and its relationship to psychopathology in children: a multimodal examination. | 2024 | Frontiers in psychiatry | Seipp V et al (Seipp et al., 2024) | Wrong outcomes. |
| Associations Between Vocal Arousal and Dyadic Coping During Couple Interactions After a Stress Induction. | 2023 | International journal of applied positive psychology | Bulling LJ (Bulling et al., 2023) | Wrong outcomes |
| Screening for Generalized Anxiety Disorder From Acoustic and Linguistic Features of Impromptu Speech: Prediction Model Evaluation Study. | 2022 | JMIR formative research | Teferra BG(Teferra et al., 2022) | Wrong design |
| Insights on Modelling Physiological, Appraisal, and Affective Indicators of Stress using Audio Features. | 2022 | Annual International Conference of the IEEE Engineering in Medicine and Biology           Society. | Triantafyllopoulos A(Triantafyllopoulos et al., 2022) | Wrong outcomes |
| Acoustic analysis of surgeons' voices to assess change in the stress response during surgical in situ simulation. | 2021 | BMJ simulation & technology enhanced learning | Hall A (Hall et al., 2021) | Wrong design |
| Measuring Stress in Health Professionals Over the Phone Using Automatic Speech Analysis During the COVID-19 Pandemic: Observational Pilot Study. | 2021 | Journal of medical Internet research | König A(König et al., 2021) | Wrong outcomes |
| Human Stress Detection: Cortisol Levels in Stressed Speakers Predict Voice-Based Judgments of Stress. | 2021 | Perception | Pisanski K(Pisanski & Sorokowski, 2021) | Same population of included study |
| Analogy instruction and speech performance under psychological stress. | 2014 | Journal of voice : official journal of the Voice Foundation | Tse AC (Tse et al., 2014) | Missing data |
| Moving beyond DSM5 and ICD11: Acoustic analysis for psychological stress on daily-wage workers in India during COVID19 | 2021 | Computers in Human Behavior Reports | Agarwal, A.(Agarwal et al., 2017) | Wrong outcomes |
| Voice fundamental frequency in the circumstances of exam stress and personality dimensions | 2012 | HealthMED | Nesic, M.(Nesic et al., 2012) | Wrong design |

## Conflict of Interest statement:

None of the authors have a conflict of interest to disclose

## Data Accessibility statement:

The data that support the findings of this study are available are available within the article and its supplementary materials. Any further data are available from the corresponding author upon reasonable request.

## Funding statement

This study received no external funding and was supported by the authors’ own means

## Ethics approval statement

This study used publicly available data and did not require approval from an ethics committee

## Patient consent statement

The research used only publicly available and anonymized data; no patient consent was needed

## Systematic Review Registration

This systematic review was registered in the PROSPERO database (registration number: CRD4202347652)

## References

Agarwal, A., Guyatt, G., & Busse, J. (2017). *Methods commentary: Risk of bias in cross-sectional surveys of attitudes and practice*. Evidence Partner. https://www.distillersr.com/resources/methodological-resources/risk-of-bias-cross-sectional-surveys-of-attitudes-and-practices

Alvear, R. M. B. De, Barón-López, F. J., Alguacil, M. D., & Dawid-Milner, M. S. (2013). Interactions between voice fundamental frequency and cardiovascular parameters. Preliminary results and physiological mechanisms. *Logopedics Phoniatrics Vocology*, *38*(2), 52–58. https://doi.org/10.3109/14015439.2012.696140

Bulling, L. J., Hilpert, P., Bertschi, I. C., Ivic, A., & Bodenmann, G. (2023). Associations Between Vocal Arousal and Dyadic Coping During Couple Interactions After a Stress Induction. *International Journal of Applied Positive Psychology*, *8*(S2), 187–204. https://doi.org/10.1007/s41042-023-00087-5

Giddens, C. L., Barron, K. W., Clark, K. F., & Warde, W. D. (2010). Beta-Adrenergic Blockade and Voice: A Double-Blind, Placebo-Controlled Trial. *Journal of Voice*, *24*(4), 477–489. https://doi.org/10.1016/j.jvoice.2008.12.002

Hall, A., Kawai, K., Graber, K., Spencer, G., Roussin, C., Weinstock, P., & Volk, M. S. (2021). Acoustic analysis of surgeons’ voices to assess change in the stress response during surgical in situ simulation. *BMJ Simulation and Technology Enhanced Learning*, *7*(6), 471–477. https://doi.org/10.1136/bmjstel-2020-000727

Kappen, M., Hoorelbeke, K., Madhu, N., Demuynck, K., & Vanderhasselt, M.-A. (2022). Speech as an indicator for psychosocial stress: A network analytic approach. *Behavior Research Methods*, *54*(2), 910–921. https://doi.org/10.3758/s13428-021-01670-x

Kappen, M., van der Donckt, J., Vanhollebeke, G., Allaert, J., Degraeve, V., Madhu, N., Van Hoecke, S., & Vanderhasselt, M. A. (2022). Acoustic speech features in social comparison: how stress impacts the way you sound. *Scientific Reports*, *12*(1). https://doi.org/10.1038/s41598-022-26375-9

Kappen, M., Vanhollebeke, G., Van Der Donckt, J., Van Hoecke, S., & Vanderhasselt, M.-A. (2024). Acoustic and prosodic speech features reflect physiological stress but not isolated negative affect: a multi-paradigm study on psychosocial stressors. *Scientific Reports*, *14*(1), 5515. https://doi.org/10.1038/s41598-024-55550-3

König, A., Riviere, K., Linz, N., Lindsay, H., Elbaum, J., Fabre, R., Derreumaux, A., & Robert, P. (2021). Measuring Stress in Health Professionals Over the Phone Using Automatic Speech Analysis During the COVID-19 Pandemic: Observational Pilot Study. *Journal of Medical Internet Research*, *23*(4), e24191. https://doi.org/10.2196/24191

Nesic, M., Cicevic, S., Nesic, V., Vuckovic, V., Kostic, J., & Manic, G. (2012). Voice fundamental frequency in the circumstances of exam stress and personality dimensions. *HealthMED - Journal of Society for Development in New Net Environment in B&H*, *6*(7), 2453–2549.

Opladen, V., Tanck, J. A., Baur, J., Hartmann, A. S., Svaldi, J., & Vocks, S. (2023). Body exposure and vocal analysis: validation of fundamental frequency as a correlate of emotional arousal and valence. *Frontiers in Psychiatry*, *14*. https://doi.org/10.3389/fpsyt.2023.1087548

Perrine, B. L., & Scherer, R. C. (2020). Aerodynamic and acoustic voice measures before and after an acute public speaking stressor. *Journal of Speech, Language, and Hearing Research*, *63*(10), 3311–3325. https://doi.org/10.1044/2020_JSLHR-19-00252

Pisanski, K., Kobylarek, A., Jakubowska, L., Nowak, J., Walter, A., Błaszczyński, K., Kasprzyk, M., Łysenko, K., Sukiennik, I., Piątek, K., Frackowiak, T., & Sorokowski, P. (2018). Multimodal stress detection: Testing for covariation in vocal, hormonal and physiological responses to Trier Social Stress Test. *Hormones and Behavior*, *106*, 52–61. https://doi.org/10.1016/j.yhbeh.2018.08.014

Pisanski, K., Nowak, J., & Sorokowski, P. (2016). Individual differences in cortisol stress response predict increases in voice pitch during exam stress. *Physiology and Behavior*, *163*, 234–238. https://doi.org/10.1016/j.physbeh.2016.05.018

Pisanski, K., & Sorokowski, P. (2021). Human Stress Detection: Cortisol Levels in Stressed Speakers Predict Voice-Based Judgments of Stress. *Perception*, *50*(1), 80–87. https://doi.org/10.1177/0301006620978378

Seipp, V., Hagelweide, K., Stark, R., Weigelt, S., Christiansen, H., Kieser, M., Otto, K., Reck, C., Steinmayr, R., Wirthwein, L., Zietlow, A., & Schwenck, C. (2024). Parenting stress in parents with and without a mental illness and its relationship to psychopathology in children: a multimodal examination. *Frontiers in Psychiatry*, *15*. https://doi.org/10.3389/fpsyt.2024.1353088

Song, Z., & Lee, P. J. (2024). Voice use of nurses working in the intensive care unit during the COVID-19 pandemic. *Intensive and Critical Care Nursing*, *82*. https://doi.org/10.1016/j.iccn.2023.103620

Teferra, B. G., Borwein, S., DeSouza, D. D., & Rose, J. (2022). Screening for Generalized Anxiety Disorder From Acoustic and Linguistic Features of Impromptu Speech: Prediction Model Evaluation Study. *JMIR Formative Research*, *6*(10), e39998. https://doi.org/10.2196/39998

Triantafyllopoulos, A., Zankert, S., Baird, A., Konzok, J., Kudielka, B. M., & Schuller, B. W. (2022). Insights on Modelling Physiological, Appraisal, and Affective Indicators of Stress using Audio Features. *2022 44th Annual International Conference of the IEEE Engineering in Medicine & Biology Society (EMBC)*, 2619–2622. https://doi.org/10.1109/EMBC48229.2022.9872012

Tse, A. C. Y., Wong, A. W.-K., Whitehill, T. L., Ma, E. P.-M., & Masters, R. S. W. (2014). Analogy Instruction and Speech Performance Under Psychological Stress. *Journal of Voice*, *28*(2), 196–202. https://doi.org/10.1016/j.jvoice.2013.03.014
